# Turbulence generation supported by an inverse energy transfer through a zig-zag pattern

**DOI:** 10.1038/s41598-026-41372-y

**Published:** 2026-02-26

**Authors:** Joel Kronborg, Johan Hoffman

**Affiliations:** KTH Royal Institute of Technology, Stockholm, Sweden

**Keywords:** Astronomy and planetary science, Physics

## Abstract

A known feature of turbulent flow in any setting, be it in ocean currents or smoke rising from a fire, is the presence of vortices on a range of scales. As turbulence develops, kinetic energy is transferred between these different scales, leading to a power law distribution of spectral energy, of a specific form established nearly a century ago. While a universally accepted mechanistic model of this process is still missing, the long-standing dominating idea is that of a turbulent energy cascade where large vortices break down into smaller ones, to successively develop finer scales until reaching a smallest scale, where energy is dissipated by viscosity. However, we here present observations of a turbulent energy spectrum developing through an alternative process. Specifically, the following problem is addressed: how is turbulence generated from the given initial condition, and what flow structures appear that may help explain the emergence of the energy spectrum? We show, using a computer simulation supported by a stability analysis, a turbulent energy spectrum emerging first at small scales and progressively extending to larger scales. This coincides in time with the formation of vortex filaments through vortex stretching on the smallest resolvable scale, and their subsequent rearrangement into recursive zig-zag patterns. It is hypothesized that the formation of this pattern leads to an inverse energy transfer from small to large scales, contributing to the development of the power law energy distribution. This description of a turbulent energy spectrum forming initially from small scales, potentially in part due to the formation of vortex filaments and their zig-zag rearrangement, rather than a forward cascade through a break-down of vortices from large scales to small, is novel to the best of our knowledge. These findings provide critical new perspectives on the development of turbulence in fluid flow, relevant in scenarios ranging from blood flow in the heart, to fuel mixing, aerodynamics, and atmospheric turbulence.

## Main

### Introduction

Turbulence is one of the unsolved mysteries of classical physics, there for everyone to observe yet elusive to explain. Often turbulence is characterized as unpredictable and chaotic, and no mathematical formula exists that describes a turbulent particle path or a turbulent velocity field. Experiments suggest that turbulence consists of complex flow structures on a range of length scales, from a macroscopic scale *L* to a microscopic scale $\eta$, the *Kolmogorov microscale*. For turbulence to develop in fluid flow, the inertial forces must dominate the viscous friction forces, corresponding to a sufficiently large *Reynolds number* $Re = UL/\nu$, where $\nu$ is the kinematic viscosity of the fluid and *U* is a characteristic velocity scale on the macroscopic length scale *L*.

If we assume a subsonic flow speed where compressibility effects may be neglected, turbulence research builds on two main results: (i) the Navier-Stokes equations^1,2^, a set of partial differential equations formulated about 200 years ago, that from given flow conditions describe the evolution of the velocity *u* and pressure *p* according to Newton’s second law and mass conservation; (ii) a statistical theory of turbulence developed almost 100 years ago^3–5^, which under the assumptions of statistical homogeneity and isotropy states that there exists an *inertial subrange* of scales $\eta< l< L$, where (in a statistical sense) velocity increments on a scale *l* satisfy a scaling law $|u(x+l)-u(x)|^2 \sim (\epsilon l)^{2/3}$ (*Kolmogorov’s 2/3 Law*), and the spectrum of kinetic energy *E*(*k*) as a function of wavenumber $k=l^{-1}$ satisfies another scaling law $E(k)\sim \epsilon ^{2/3}k^{-5/3}$ (*Kolmogorov’s −5/3 Law*).

One fundamental assumption of the statistical theory is that the spectral energy flux in the inertial subrange is equal to the mean energy dissipation rate per unit mass $\epsilon$. Another fundamental assumption is that viscous dissipation only plays a role at the Kolmogorov microscale $\eta$. As long as these two assumptions hold, the scaling laws follow from a simple dimensional analysis. With $\epsilon$ constant (in a statistical sense), these scaling laws also express a *scale similarity* of turbulence. Further, the statistical theory predicts that $\eta /L \sim Re^{-3/4}$ and $u_{\eta }/U \sim Re^{-1/4}$, where $u_{\eta }$ is a charactieristic velocity scale at $\eta$.

In time, the statistical theory was refined to reflect the fact that energy dissipation is more localized than suggested by the original theory^6,7^, which also lead to the development of *multifractal models* to capture this *intermittency* of turbulence^8^. But the precise nature of this process, the *turbulent energy cascade*, remains an open problem, which we address in this article.

### The turbulent energy cascade

In an often cited poem from 1922^9^, Lewis F. Richardson formulated his intuitive idea about the cascade as a hierarchical distribution of vortices down to a viscous scale:

Big whirls have little whirls that feed on their velocity,
And little whirls have lesser whirls and so on to viscosity

Richardson’s idea has largely remained as a qualitative description of the cascade, but a universally accepted, mechanistic model for how such a distribution of vortices would emerge in the inertial subrange is still missing. Taylor and Green in 1937^10^ introduced an explicit analytical example of a set of periodic vortices, for which they showed how the non-linear term of the Navier-Stokes equations could transfer energy to smaller vortices of a similar type. In 1938^11^, Taylor then suggested that the primary mechanism for the cascade is vortex stretching, where straining flow in the direction of the vortex axis leads to an intensification of the vortex. Vortex stretching has remained as a dominating idea of the cascade^12^, more recently in combination with the process of reconnection of vortex filaments of different shapes and topologies^13^. Where inverse energy transfer patterns have been observed in vortex reconnection scenarios, e.g. by Yao & Hussain^14^, it has mainly been viewed as a peripheral phenomenon attributed to the merging of small adjacent vortices. In 2D turbulence, the inverse cascade is a well-known phenomenon^15^, and it has previously shown to be possible in slightly modified Navier-Stokes equations in 3D^16^. Inverse energy transfer is also an established phenomenon in boundary layers in wall-bounded flow^17^.

In 2020, McKeown et al.^18^ proposed a model of the cascade as an iterated *elliptic instability*, based on a scenario where two counter-rotating vortices in close proximity generate an array of new perpendicular, pairwise counter-rotating vortices on a finer scale. Their model of the cascade is based on the idea that the same scenario can be recursively repeated for the new vortices on the finer scale, and so on, until reaching the Kolmogorov microscale. While this model echoes the intuitive idea of Richardson, it also provides a concrete mechanism for how *big whirls could generate little whirls, that in turn could generate lesser whirls*.

In this article, we present the emergence of a turbulent energy spectrum through an alternative pathway, supported by our previous work on a mathematical stability analysis of the Navier-Stokes equations^19^, in computer simulations of developing turbulent flow. Our model is related to the scenario of McKeown et al., but with two key differences: First, starting from two counter-rotating vortices, in our simulations we can reproduce the generation of an array of perpendicular, pairwise counter-rotating vortices on a finer scale, *but with the key difference that this finer scale is the finest scale possible*, the Kolmogorov microscale $\eta$. As these vortex filaments form, there is an early rise in energy at high *k*, corresponding to the small length scale, and the Kolmogorov $-5/3$ Law subsequently develops dynamically from high *k* to low. With the focus of the study being flow patterns in emerging turbulence, rather than fully developed turbulence, the simulation case is neither fully homogeneous nor isotropic. The advantage of these simulations, however, is that they enable identification of specific features in the flow, that can be linked to the emerging $-5/3$ slope in the energy spectrum, as the global flow evolves to a progressively more turbulent state.

**Fig. 1:**
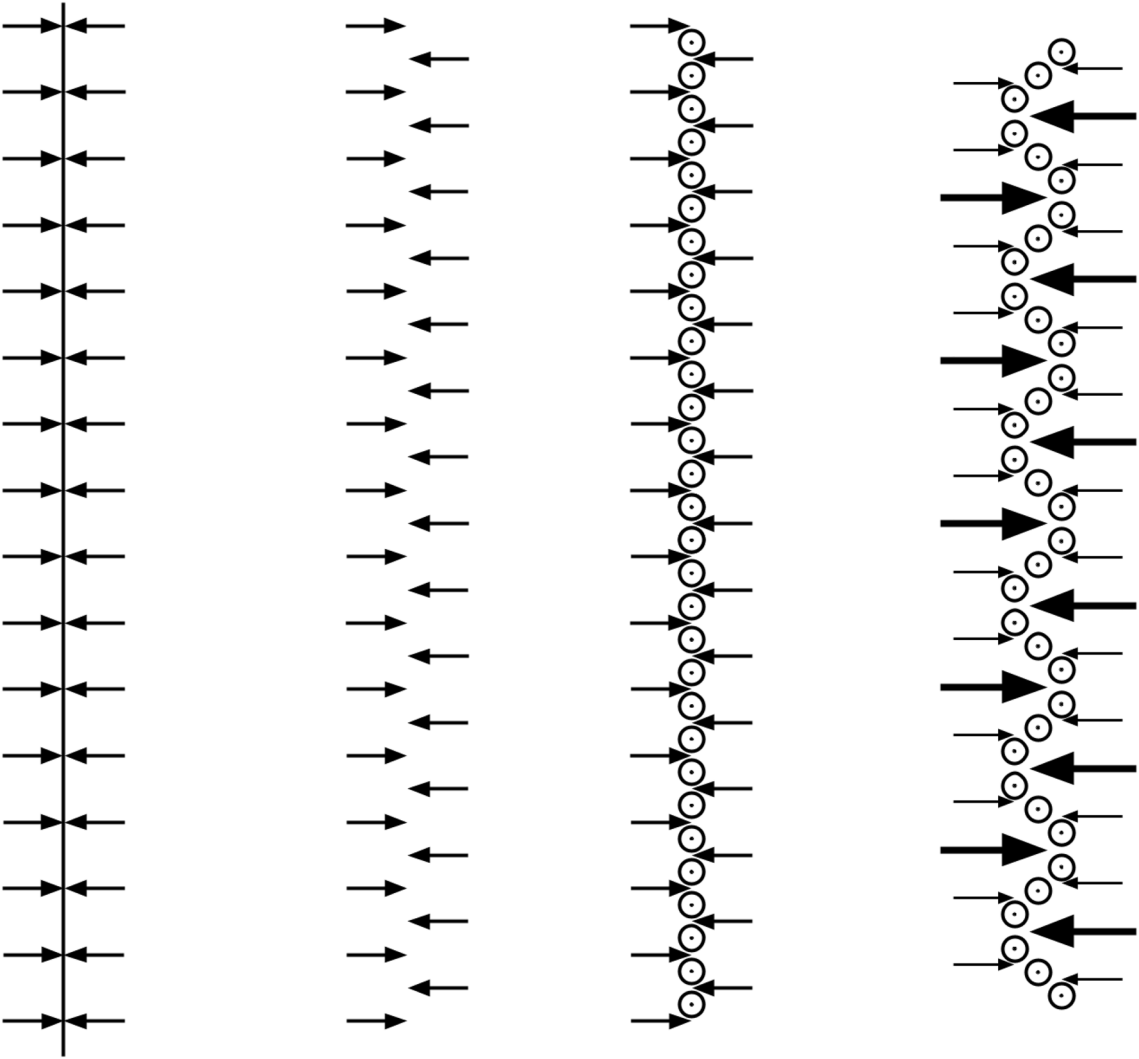
Illustration of the zig-zag pattern. Evolving from left to right: a cut through a strain field with stretching flow in the out-of-paper direction and compressing flow in the cut plane towards a center line; small perturbations on the finest scale possible; generation of an array of pairwise, counter-rotating vortices on the microscale in the out-of-paper direction; zig-zag pattern emerging in which every third vortex pair (in each direction) dominates.

We hypothesize that the emergence of the turbulent energy spectrum in this simulation is partly driven by an inverse energy transfer arising from interactions between the vortex filaments. This interpretation is supported by the observation that the micro-vortices reorganize in a zig-zag pattern, as a consequence of small perturbations in their positions, causing certain vortex pairs to dominate their neighbors. This reorganization coincides in time with increasing energy at larger length scales. Since the zig-zag pattern effectively corresponds to a relative rotational motion of the vortex filaments, it is plausible that this process contributes to the rise in energy at lower wavenumbers. This process is then recursively repeated to develop a full inertial range of scales. In Fig. 1, we sketch the first steps of this process, where an array of counter-rotating micro-vortices are generated in a strain field, which then develops into a zig-zag pattern.

We next present a mathematical analysis of the Navier-Stokes equations, consisting of two main building blocks: the triple decomposition of the velocity gradient tensor^20^, and a novel stability analysis using this idea^19^. The triple decomposition is used to visualize and identify specific flow structures in the simulation, while the stability analysis provides analytical expectations for the evolution and stability of the observed flow structures.

### Triple decomposition of the velocity gradient tensor

With $u=(u_1,u_2,u_3)$ the flow velocity vector defined at a spatial point $x=(x_1,x_2,x_3)$ at time *t*, the velocity gradient tensor $\nabla u$ characterizes the spatial structure of the velocity field near *x* at time *t*.

In three dimensions, the velocity gradient tensor can be represented by a $3\times 3$ matrix, with a standardized real Schur form

1 $$\begin{aligned} \nabla u= Q^T \overline{\nabla u}\, Q, \end{aligned}$$

where *Q* is an orthogonal matrix corresponding to a change of basis, and the superscript *T* represents the transpose of a matrix. If $\nabla u$ only has real eigenvalues, then $\overline{\nabla u}$ is an upper triangular matrix, and if it is also a normal matrix, meaning that $\nabla u^T \nabla u=\nabla u\nabla u^T$, then $\overline{\nabla u}$ is diagonal.

On the other hand, if $\nabla u$ has complex eigenvalues, which necessarily appear as a complex conjugate pair, then $\overline{\nabla u}$ is a quasi-upper triangular matrix with a $2\times 2$ block on the diagonal. If we choose *Q* such that the $2\times 2$ block has identical diagonal entries, referred to as the Schur form being standardized^21^, and is positioned in the lower right corner, then the representation is unique (modulo certain sign changes) and takes the form^20^:

2 $$\begin{aligned} \overline{\nabla u} = \left[ \begin{array}{ccc} \lambda _1 & \epsilon & \zeta \\ 0 & \alpha & \beta \\ 0 & \gamma & \alpha \end{array} \right] = \left[ \begin{array}{ccc} \frac{\partial \overline{u}_1}{\partial \overline{x_1}} & \frac{\partial \overline{u}_1}{\partial \overline{x_2}} & \frac{\partial \overline{u}_1}{\partial \overline{x_3}} \\ 0 & \frac{\partial \overline{u}_2}{\partial \overline{x_2}} & \frac{\partial \overline{u}_2}{\partial \overline{x_3}} \\ 0 & \frac{\partial \overline{u}_3}{\partial \overline{x_2}} & \frac{\partial \overline{u}_3}{\partial \overline{x_3}} \end{array} \right] , \end{aligned}$$

where $\overline{u}=(\overline{u}_1,\overline{u}_2,\overline{u}_3)$ is the velocity vector field expressed in the new coordinates $\overline{x}=(\overline{x}_1,\overline{x}_2,\overline{x}_3)$, defined by the orthogonal matrix *Q*.

We can now decompose the tensor $\overline{\nabla u}$ into a sum of three tensors,

3 $$\begin{aligned} \overline{\nabla u} = \overline{\nabla u}_{ST} + \overline{\nabla u}_{SH} + \overline{\nabla u}_{RR}, \end{aligned}$$

defined as

4 $$\begin{aligned} \overline{\nabla u}_{ST} = \left[ \begin{array}{ccc} \lambda _1 & 0 & 0 \\ 0 & \alpha & 0 \\ 0 & 0 & \alpha \end{array} \right] , \end{aligned}$$

5 $$\begin{aligned} \overline{\nabla u}_{SH} = \left[ \begin{array}{ccc} 0 & \epsilon & \zeta \\ 0 & 0 & \textrm{sgn}(\beta )\max (\vert \beta \vert - \vert \gamma \vert ,0) \\ 0 & \textrm{sgn}(\gamma )\max (\vert \gamma \vert - \vert \beta \vert ,0) & 0 \end{array} \right] , \end{aligned}$$

6 $$\begin{aligned} \overline{\nabla u}_{RR} = \left[ \begin{array}{ccc} 0 & 0 & 0 \\ 0 & 0 & \textrm{sgn}(\beta )\min (\vert \beta \vert , \vert \gamma \vert ) \\ 0 & \textrm{sgn}(\gamma )\min (\vert \beta \vert , \vert \gamma \vert ) & 0 \end{array} \right] , \end{aligned}$$

where we note that $\overline{\nabla u}_{ST}$ is a diagonal matrix, $\overline{\nabla u}_{SH}$ is a non-normal strictly upper triangular matrix (assuming that $\vert \beta \vert \ge \vert \gamma \vert$), and $\overline{\nabla u}_{RR}$ is a skew-symmetric matrix. The *triple decomposition* of the velocity gradient tensor is then defined as

7 $$\begin{aligned} \nabla u= Q^T \overline{\nabla u}_{ST}\, Q + Q^T \overline{\nabla u}_{SH}\, Q + Q^T \overline{\nabla u}_{RR}\, Q. \end{aligned}$$

The Schur form is a purely algebraic property of the velocity gradient tensor as a $3\times 3$ matrix, but the triple decomposition has also a very specific mechanical interpretation, where each term in the sum is associated with a fundamental flow structure: $\overline{\nabla u}_{ST}$ is a pure strain rate tensor, $\overline{\nabla u}_{SH}$ is a shear rate tensor, and $\overline{\nabla u}_{RR}$ is a rigid body rotation rate tensor. Euler’s rotation theorem^22^ states that rigid body rotation defines a unique rotation axis, and the frame of reference defined by *Q* is aligned with this unique rotation axis, given by the first column vector of $\overline{\nabla u}$, expressed as $(\lambda _1,0,0)$ in the new coordinates $\overline{x}$^20^.

### Stability analysis of the Navier-Stokes equations

Hydrodynamic stability can be framed as the question of perturbation growth in a given flow configuration governed by the Navier-Stokes equations,

8 $$\begin{aligned} \begin{array}{c} \displaystyle {\frac{\partial u}{\partial t}} + (u\cdot \nabla ) u + \nabla p - \nu \Delta u = 0, \\ \nabla \cdot u = 0, \end{array} \end{aligned}$$

here expressed using standard notation for the differential operators. Given initial and boundary conditions, the Navier-Stokes equations describe the evolution of fluid flow. The first equation is Newton’s second law, and the second equation is the incompressibility constraint, corresponding to mass conservation. For simplicity, we here assume that all external forces (like gravity) are zero.

The total energy of a velocity perturbation $\varphi =(\varphi _1,\varphi _2,\varphi _3)$ is expressed as the integral of $1/2\vert \varphi \vert ^2=1/2(\vert \varphi _1\vert ^2+\vert \varphi _2\vert ^2+\vert \varphi _3\vert ^2)$ over the spatial domain of the flow $\Omega \subset \mathbb {R}^3$, and the Navier-Stokes equations can be used to derive an expression for the time evolution of this energy^19^,

9 $$\begin{aligned} \frac{d}{dt} \int _{\Omega } \frac{1}{2} \vert \varphi \vert ^2\, dx = - \int _{\Omega } \varphi ^T \nabla u \, \varphi \, dx - \int _{\Omega } \nu \vert \nabla \varphi \vert ^2\, dx, \end{aligned}$$

where the first term on the right-hand side represents potential growth or decay of the perturbation energy, and the second term corresponds to damping of the perturbation energy by viscous dissipation.

The assumption that viscosity only plays a role at the Kolmogorov microscale implies that we can neglect viscous damping when we study perturbations on larger scales. Using the triple decomposition, we can then approximate the evolution of perturbation energy in turbulent flow on coarser scales than the Kolmogorov microscale as:

10 $$\begin{aligned} \frac{d}{dt} \int _{\Omega } \frac{1}{2} \vert \varphi \vert ^2\, dx \approx - \int _{\Omega } \varphi ^T \nabla u \, \varphi \, dx = - \int _{\Omega } (Q \varphi )^T (\overline{\nabla u}_{ST} + \overline{\nabla u}_{SH})Q\varphi \, dx, \end{aligned}$$

where we have used that $\overline{\nabla u}_{RR}$ is a skew-symmetric tensor, therefore,

11 $$\begin{aligned} \int _{\Omega } (Q \varphi )^T \overline{\nabla u}_{RR} \, Q\varphi \, dx = 0. \end{aligned}$$

Hence, whether the energy of perturbations will grow or decay is determined by the flow structures of strain and shear, but in very different ways. The strain tensor is diagonal, which corresponds to exponential growth or decay in time, depending on the signs of the entries $\lambda _1$ and $\alpha$. The incompressibility constraint of the Navier-Stokes equations is equivalent to the trace of the velocity gradient tensor being zero, which leads to the conclusion that $\lambda _1+2\alpha =0$. That is, if strain is non-zero, there will always be exponential growth of perturbations, either in the direction of $\lambda _1$, or in the orthogonal plane. Perturbation growth in shear flow is polynomial (linear or quadratic) in time, a consequence of the non-normal shear rate tensor $\overline{\nabla u}_{SH}$, which is well known, e.g., in the study of transition to turbulence^23–26^. Finally, rigid body rotational flow is stable in time, since no perturbation growth is induced by the skew-symmetric tensor $\overline{\nabla u}_{RR}$.

A complete turbulent flow field is of course a combination of strain, shear and rotation, but the stability analysis above describes the local perturbation growth depending on which fundamental flow structure that dominates. This suggests a characterization of turbulent flow as a combination of stable rotational flow structures (vortex filaments) that can exist for a long time, unstable shear flow structures (shear layers and shear pancakes) that exist for a short time before transforming into stable rotational flow structures (shear layer/pancake roll-up into vortex filaments), and sudden bursts of exponentially unstable straining flow structures that generate or intensify vortex filaments and/or shear layers/pancakes.

The nature of turbulence is also studied through the vorticity field, which is defined as the curl of the velocity field $\omega = \nabla \times u$. Vorticity can represent either rotation or shear, or a combination thereof. A stability analysis analogous to the above shows that enstrophy exhibits exponential growth through strain and linear/quadratic growth through shear^19^,

12 $$\begin{aligned} \frac{d}{dt} \int _{\Omega } \frac{1}{2} \vert \omega \vert ^2\, dx = \int _{\Omega } (Q \omega )^T (\overline{\nabla u}_{ST} + \overline{\nabla u}_{SH})Q\omega \, dx - \int _{\Omega } \nu \vert \nabla \omega \vert ^2\, dx . \end{aligned}$$

More specifically, if $\lambda _1>0$, then vorticity in the form of rotation about the rotation axis grows exponentially, known as vortex stretching. If $\lambda _1<0$, vorticity grows exponentially across the plane orthogonal to the rotation axis, in the form of sharpening of a shear layer or a shear pancake. However, note that nothing in this analysis excludes that shear grows also if $\lambda _1>0$, which we observe in our computer simulations.

Having outlined the triple decomposition as a means of visualizing and identifying flow structures in the simulation, and the stability analysis which provides expectations for the stability of rotational and strain-dominated structures, the discussion now turns to the computer simulations. With the provided framework in mind, we examine how turbulence develops in the flow and assess the extent to which the observed dynamics agree with the theoretical expectations.

### Computer simulation

We now revisit a scenario similar to that of McKeown et al.^18^, in the form of a computer simulation of a pair of adjacent counter-rotating vortices of Taylor-Green type^10^, oriented in the $x_1$-direction and aligned in the $x_2$-direction, initialized in a box of size $3\times 4\times 8$ (in a non-dimensional length unit) with slip boundary conditions on the walls, i.e., the wall-normal velocity is set to zero. We use a stabilized finite element method to compute approximate solutions to the Navier-Stokes equations on a structured tetrahedral mesh of $72 \times 10^6$ piecewise linear elements, with a second order accurate trapezoidal time stepping method.

The viscosity $\nu$ is set to zero to model a very high Reynolds number, but energy is still dissipated through numerical dissipation of the numerical method, and an effective Kolmogorov microscale $\eta _h \sim h$ is induced by the stabilization of the numerical method, with a mesh resolution $h = 0.035$. This type of turbulence simulation is well established, referred to as an implicit large eddy simulation, or residual-based turbulence modeling^27–34^.

**Fig. 2:**
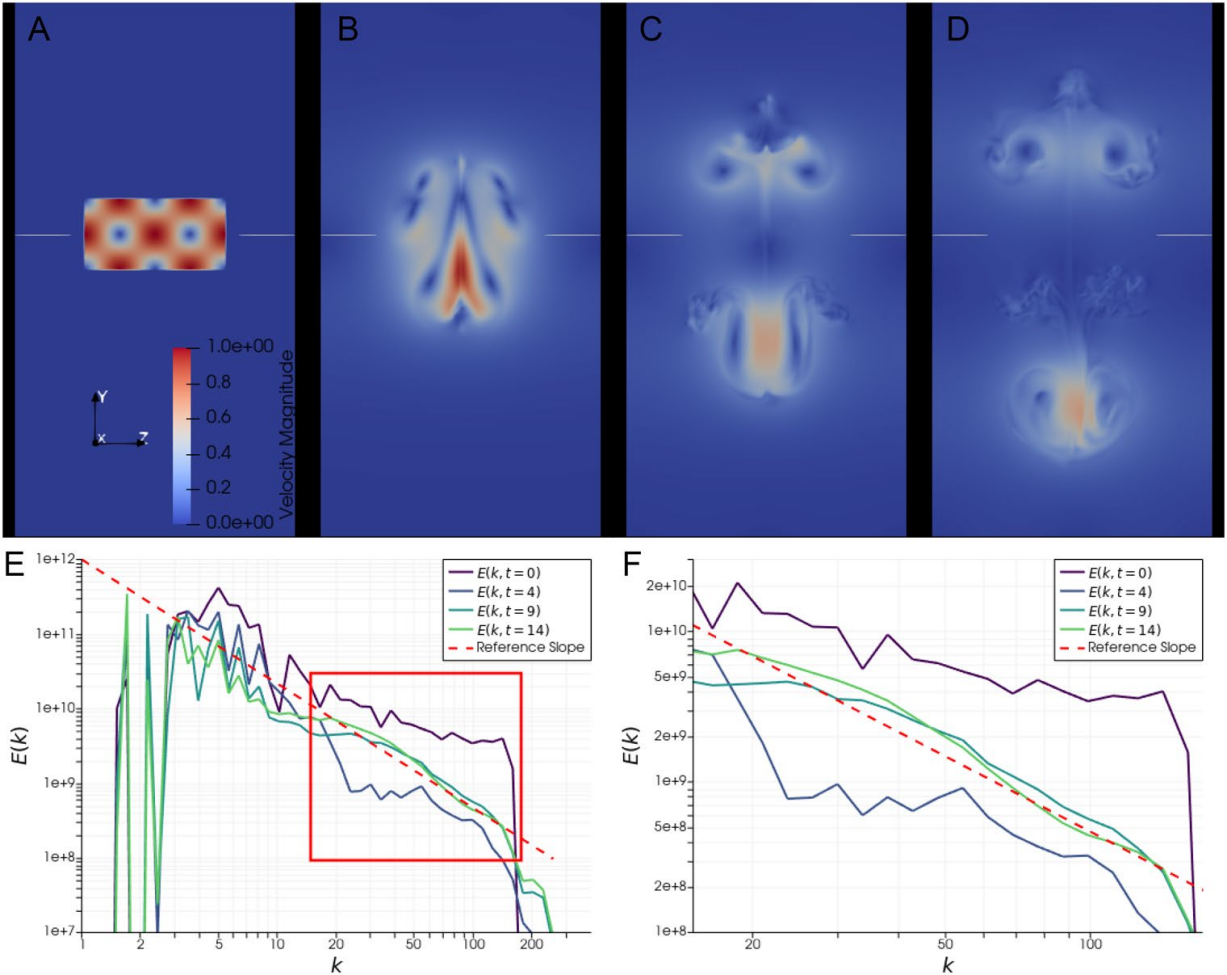
Velocity fields and energy spectrum. (**A**–**D**) Velocity magnitude in a vertical cut through the domain at times $t=[0,4,9,14]$. The initial pair of vortices breaks up into two secondary pairs of vortices, one pair moving up and one pair moving down. The horizontal grey lines mark the location of the horizontal cut used in Fig. 3. (**E**,**F**) Energy spectrum of the velocity field computed using a Fourier transform of *u*, plotted against $|\vec {k}|$, at times corresponding to the snapshots. The reference slope is given by the power law $|\vec {k}|^{-5/3}$, which is the expected energy distribution in turbulent flow. At $t=9$ and $t=14$ the energy spectrum converges to this slope for $|\vec {k}|\in [20,140]$, which is consistent with the emergence of turbulence and an intertial subrange. This area is marked by the red rectangle in (**E**), and shown in detail in (**F**).

Snapshots of the velocity field in the simulation are displayed in Fig. 2A–D. The initial pair of vortices can be observed to split up into two pairs of secondary vortices, one pair moving up and one pair moving down in the domain. We also see that with time, the energy spectrum in Fig. 2E,F approaches the characteristic −5/3 slope postulated by Kolmogorov, indicating that turbulence with an inertial range has indeed developed. To link the spectrum to clearly distinguishable features in the flow, which enables us to address the question of how turbulence is generated from this initial condition, we first consider the energy spectrum in a subdomain of the simulation.

**Fig. 3:**
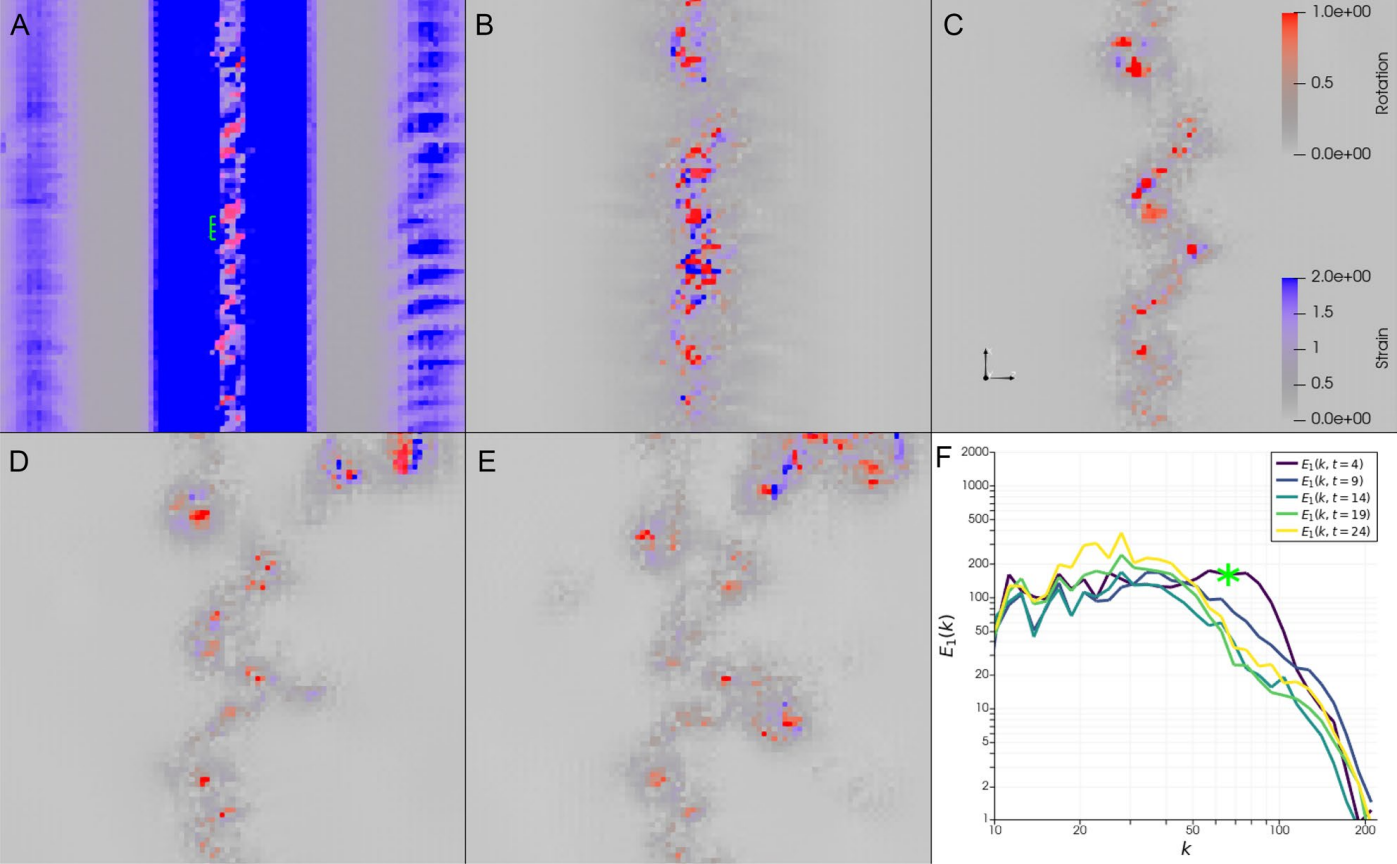
Local zig-zag pattern and energy spectrum. (**A**–**E**) Snapshots of rotation (red) and strain (blue) in a horizontal cut through the domain at the height marked in grey in Fig. 2A–D, taken at times $t=[4,9,14,19,24]$. Areas with rotation where $||\nabla u_{RR}||>=1$ and strain where $||\nabla u_{ST}||>=2.0$ are colored as solid red and blue, respectively, and lower values as successively less opaque. Vortices at the smallest resolvable scale first appear along a straight line at the center of the strain field (A), and later arrange themselves in a zig-zag pattern as the strain decays (**C**–**E**). (**F**) Energy spectrum of the velocity field computed using a Fourier transform of $u_1$, plotted against $|\vec {k}|$, at times corresponding to the snapshots. The green asterisk marks the value of $|\vec {k}|$ that corresponds to the length marked in green in (A).

The chosen subdomain is displayed in Fig. 3, in the form of a 2D horizontal plane positioned between the two pairs of secondary vortices. The location of this 2D plane is also marked by the grey horizontal lines in Fig. 2A–D. Figure 3 shows snapshots of the Frobenius norms of rotation and strain from the triple decomposition at five equally spaced time steps, as well as the energy spectrum of the velocity component $u_1$ for those time steps (selected to highlight the transfer of energy from the microscale $\eta _h$). Initially, at $t=4$, a strain field induces an array of vortices (visible as red spots in Fig. 3A) on the microscale $\eta _h$, perpendicular to the plane, and spaced along a line in the $x_1$-direction. As the strain field rapidly diminishes the vortices remain (Fig. 3B). Note that this is in accordance with the stability analysis of strain (exponentially unstable) and rotation (stable). As the flow evolves, the vortices stray from their original alignment to instead arrange themselves in a zig-zag pattern, clearly visible at $t=14$ (Fig. 3C). The similarity to the idealized sketch in Fig. 1 is apparent. The zig-zag pattern corresponds to the development of rotational structures on a coarser scale, whose effect can be observed at $t=19$ and $t=24$ (Fig. 3D,E). In the last two snapshots, flow features originating from other subdomains in the flow have also started to appear, particularly in the top right corner.

The energy spectrum (Fig. 3F) for the snapshot at $t=4$ shows a peak at high wave numbers, from $k\approx 55$ to $k\approx 75$. This corresponds to length scales in the range [0.08, 0.11], which is approximately the length between adjacent vortices that have formed, as indicated by the green line in Fig. 3A. At $t=9$ this peak has diminished, and at the following time snapshots the spectrum converges to an approximately linear slope in the log-log plot for *k* in the range [40, 140], which corresponds to lengths in the range [0.04, 0.16]. Interestingly, although the peak in the energy spectrum shifts to lower wave numbers after the initial time, the size of the vortices does not change drastically. Instead, the transfer of energy to larger scales appears to correspond to a new period emerging in the zig-zag pattern the vortices align themselves in. This marks a significant departure from the conventional view, which has long held that energy scales are represented by the size of individual vortices, rather than their motion relative to each other.

**Fig. 4:**
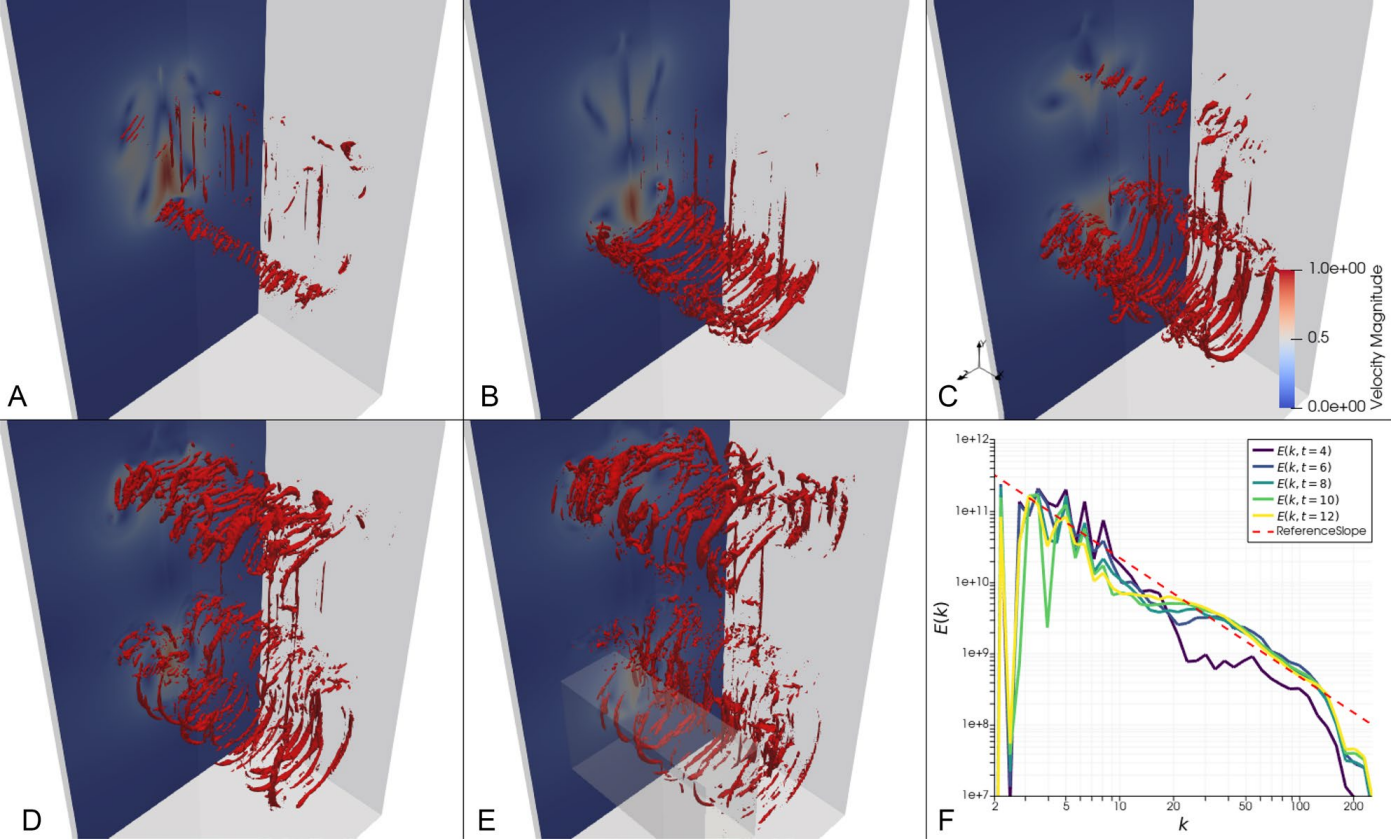
Rotation structures and energy spectrum. (**A**–**E**) Snapshots of a vertical cut through the velocity field (back) and 3D rotation structures in red. The rotation structures shown have $||\nabla u_{RR}||>=1.5$ (A) or $||\nabla u_{RR}||>=2$ (**B**–**E**), and are shown at times $t=[4,6,8,10,12]$. The large grey box outlines the simulation domain, and the small grey box in (E) marks the subdomain depicted in detail in Fig. 5. (**F**) Energy spectrum of the velocity field computed using a Fourier transform of *u*, plotted against $|\vec {k}|$, at times corresponding to the snapshots. The reference slope is given by the power law $|\vec {k}|^{-5/3}$, which is the expected energy distribution in turbulent flow.

With these local observations in mind, we return to study the global flow. Figure 4A–E displays snapshots of high rotation areas colored as red, next to planar velocity fields at the same points in time. Additionally, Figure 4F shows the total energy spectrum of the entire domain at the same instants. At $t=4$ (Fig. 4A), some of the longitudinal vortices on the smallest resolvable scale, featured in Fig. 3, are clearly visible centrally. As time progresses, similar small scale vortices are first generated around the bottom of the lower pair of secondary vortices (Fig. 4B,C), and then around the top of the upper pair of secondary vortices (Fig. 4D,E).

In the energy spectrum (Fig. 4F), for $t=4$ the energy is approximately uniform between $k=24$ and $k=54$, and for higher *k* follows roughly the $-5/3$ reference slope until $k=110$. For higher *t*, the energy level rises and converges to the $-5/3$ slope between $k=20$ and $k=140$, with the part at the lowest of these *k* values being the slowest to converge to the slope. From these results alone, it is not evident whether the energy spectrum in the inertial range develops exclusively through an energy transfer from larger scales. It is plausible that an inverse energy transfer occurs that contributes to the development of the slope, again originating from the small scale vortex filaments rearranging in zig-zag patterns.

We can now propose a mechanistic description through the stability analysis above for this scenario: (1) two stable adjacent anti-parallel vortices on the macroscopic scale *L* are close enough to establish a perpendicular strain field; (2) this exponentially unstable strain field induces an array of stable, pairwise counter-rotating vortex filaments on the microscale $\eta _h$, through vortex stretching originating from perturbations in the flow; (3) the stable microscopic vortex filaments are rearranged into a zig-zag pattern which results in a transfer of energy from $\eta _h$ to larger scales $l>\eta _h$, contributing to the emergence of the Kolmogorov $-5/3$ Law in an inertial subrange of scales between *L* and $\eta _h$.

While the first step (1) is close to the scenario described in the work of McKeown et al.^18^, the second step (2) differs in that the array of vortices is generated at the Kolmogorov microscale, and the third step (3) is completely novel as far as we are aware, and provides a complement to established ideas of turbulence developing exclusively through an energy cascade from large scales to small.

**Fig. 5:**
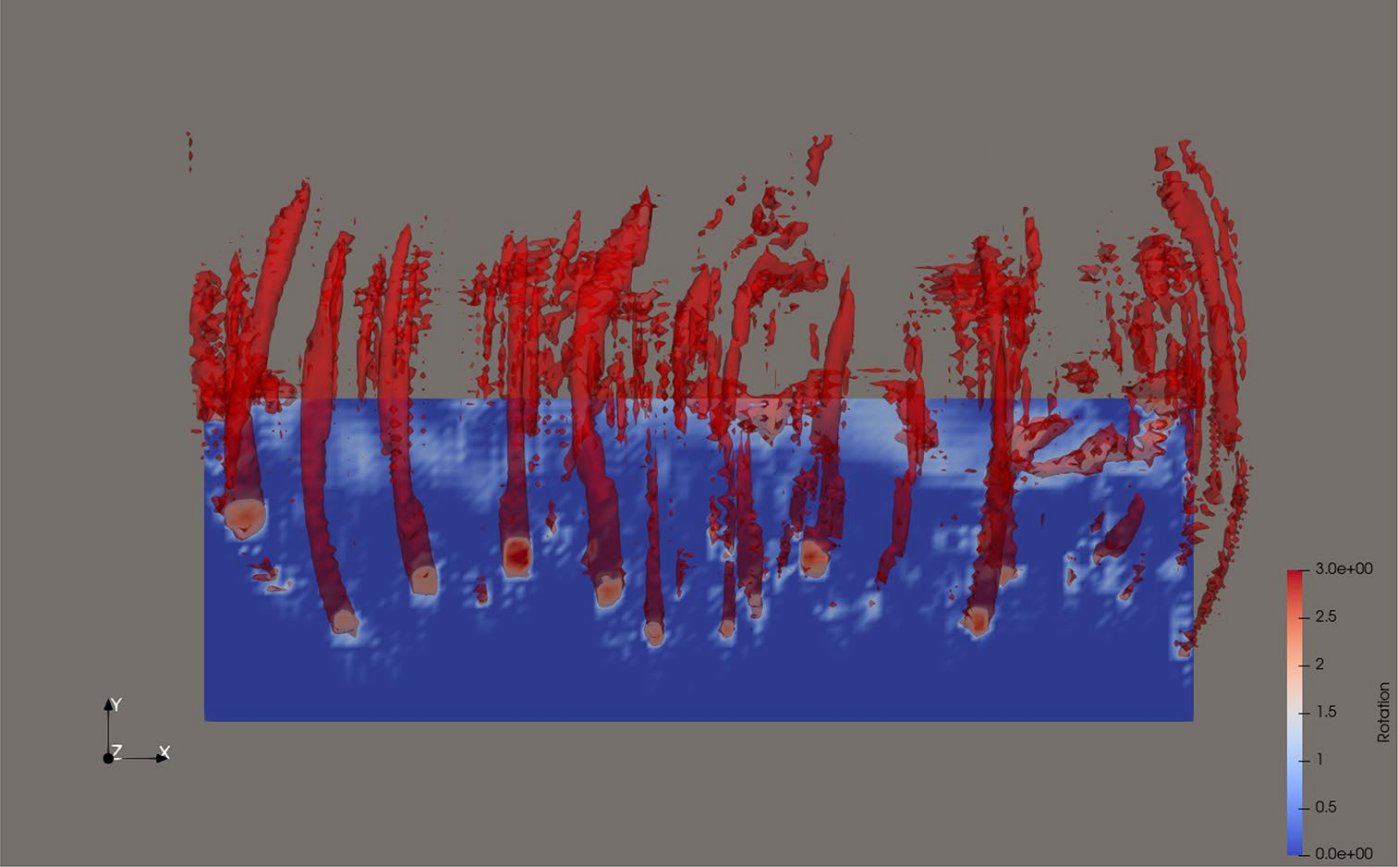
Zig-zag pattern around large vortices. An example of the zig-zag pattern appearing for small vortices around one of the large vortices at time $t=12$. The small vortices are shown in the forefront in semi-transparent red. Behind them is a vertical cut through the rotation field, which more clearly shows the zig-zag pattern in this plane.

The connection between the development of the energy spectrum and the prevalent vortex structures is naturally not as straight-forward in the full domain in Fig. 4 as it is in the local subdomain in Fig. 3, as the structures are more numerous as well as more complex. But a qualitative observation of the structures still suggests that the turbulent energy spectrum also in the full domain is not developed exclusively as the result of large scale vortices cascading down to successively smaller vortices in the inertial range, to eventually reach the Kolmogorov microscale. The turbulent energy spectrum emerges initially for the higher *k* in the inertial range, and then expands to lower *k*, potentially supported by an inverse energy transfer. This happens not primarily as a result of small vortices connecting to form larger ones, as is clear in Fig. 4 A–E. Instead, by examining details of how the small scale vortices rearrange their positions relative to each other in different parts of the domain, an example of which is displayed in Fig. 5, we see the zig-zag pattern appearing once again. This indicates that the generation of turbulence in this scenario is supported by a process of first formation of small scale vortices, and then the rearranging of these vortices in a zig-zag pattern.

As an idealized illustration of the emerging zig-zag pattern, consider an array of out of the plane, pairwise counter-rotating vortices at a uniform distance *d*, embedded in a strain field which stretches the vortices out of the plane and generates a compressing flow in the plane which flows through the array in alternating directions, cf. Fig. 1. Now, assume that the center of one vortex is perturbed along the array by a small perturbation $\delta>0$, so that the distance to the vortex on one side is $d-\delta$ and on the other side it is $d+\delta$. This will result in a lower mass flow on the first side of the center, and a higher mass flow on the other side of the center, which in turn will generate shear that will deform and move the vortex relative to the neighboring vortices. With similar perturbations of alternating signs for every other vortex, the induced shear will deform the array into a zig-zag pattern, albeit in practice somewhat more irregular than what is described here.

As observed above, the zig-zag pattern corresponds to rotation on a larger scale, which can recursively propagate to transfer spectral energy to larger scales in a self-similar process.

In conclusion, since the assumption that the turbulent energy cascade forms as a result of large vortices breaking down to form new vortices on smaller scales has been central for decades, the demonstration of a potential contribution from an inverse energy transfer opens up for new insights. The zig-zag pattern also gives a potential mechanism for self-similarity in turbulence. Ultimately, these findings may have important implications for turbulence research, including new interpretations of existing experimental and computer simulation results, as well as high impact for applied research in a range of areas.

## Methods

### Stability of flow structures

Detailed descriptions of the triple decomposition of the velocity gradient tensor and the stability analysis of the Navier-Stokes equations can be found in our previous work^19,20^. One key step of the stability analysis is to show that perturbation energy does not grow in pure rotational flow, which follows from the fact that the quadratic form $v^TAv$ of any skew-symmetric matrix *A* is zero, since $A^T=-A$, and, therefore, we have that

$$\begin{aligned} v^TAv = (Av)^Tv = v^TA^Tv = - v^TAv, \end{aligned}$$

which proves that $v^TAv=0$. Hence, for $A=\overline{\nabla u_{RR}}$ and $v=Q\varphi$, equation (11) follows.

Exponential growth of perturbations in pure straining flow is a consequence of the scalar ordinary differential equation

13 $$\begin{aligned} \dot{v}(t) = a v(t), \end{aligned}$$

with solution $v(t) = v(0)\exp (at)$. Since the matrix $\overline{\nabla u_{ST}}$ is diagonal, the vector equation for the velocity perturbation decouples into three scalar equations in the form of equation (13), with solutions

$$\begin{aligned} (Q\varphi )_1(t) = (Q\varphi )_1 (0)\exp (\lambda _1 t), \quad (Q\varphi )_i(t) = (Q\varphi )_i (0)\exp (\alpha t),\quad i=2,3. \end{aligned}$$

The strictly upper triangular form of $\overline{\nabla u_{SH}}$ gives by simple integration that perturbation growth in $(Q\varphi )_3$ is zero, in $(Q\varphi )_2$ linear in time, and in $(Q\varphi )_1$ quadratic in time, assuming that $\epsilon ,\zeta \ne 0$ and $\vert \gamma \vert \ne \vert \beta \vert$.

### Computer simulation of turbulent flow

To study turbulence we compute solutions to the incompressible Euler equations, using a stabilized Galerkin finite element method. We use no turbulence model other than the numerical dissipation introduced by the stabilization of the numerical method, corresponding to an implicit large eddy simulation (ILES) or residual-based turbulence modeling^27,28,31^. Continuous linear finite element basis functions were used on a structured mesh of $72 \times 10^6$ tetrahedrons, together with a second order accurate trapezoidal time stepping method. The resolution of the mesh, together with the dissipative effect of the stabilization, induces a finest simulation scale $\eta _h$, which corresponds to an effective Kolmogorov microscale in the simulations.

More specifically, the simulations are based on the incompressible Euler equations,

14 $$\begin{aligned} \begin{array}{c} \displaystyle {\frac{\partial u}{\partial t}} + (u\cdot \nabla ) u + \nabla p = 0, \\ \nabla \cdot u = 0, \end{array} \end{aligned}$$

starting from an initial velocity field,

15 $$\begin{aligned} u=u(x)=(u_1(x),u_2(x),u_3(x)),\quad x = (x_1,x_2,x_3), \end{aligned}$$

based on the Taylor-Green vortex solutions^10^,

16 $$\begin{aligned} \begin{array}{ccc} u_1(x) & =& 0, \\ u_2(x) & =& \cos (\pi (x_2-1/2))\sin (\pi (x_3-1/2)), \\ u_3(x) & =& \sin (\pi (x_2-1/2))\cos (\pi (x_3-1/2)), \end{array} \end{aligned}$$

but with the difference that only one pair of counter-rotating vortices are used, corresponding to the conditions that $0 \le x_2 \le 1$ and $-1 \le x_3 \le 1$. The domain is a box of size $3\times 4\times 8$ in a non-dimensional length unit, and slip boundary conditions are used (wall-normal velocity is set to zero).

This turbulence simulation method has been tested and validated for a range of applications^29–34^. Being an ILES method, the finest scale in the simulation is given by the mesh resolution. In physical terms, an ILES simulation on a coarser mesh corresponds to a scenario with a lower Reynolds number, coarser Kolmogorov microscale, and not as wide inertial subrange.

**Fig. 6:**
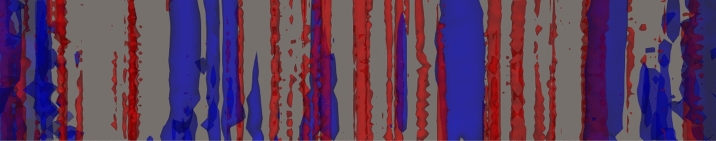
Mesh resolution comparison. Snapshot at $t=11$ of the small central vortices with two different mesh resolutions. The red vortices are on the fine mesh featured in the rest of this study, whereas the blue vortices are on a coarser mesh for comparison.

To examine the effect of the mesh resolution in our simulation and to verify the emergence of vortices on the smallest resolvable scale, an additional simulation was run with the same boundary and initial conditions, but on a coarser mesh. The coarser mesh consisted of $9\times 10^6$ tetrahedrons, which corresponds to a cell diameter of $h_{coarse} = 2h = 0.069$. Figure 6 shows a detail snapshot of central small scale vortices, at an early stage of the zig-zag pattern development, on both the coarse mesh and the fine mesh used in the study. The image confirms qualitatively similar results on the coarser mesh, with the formation of vortices on a slightly larger scale due to the lower resolution, and with longer distance between each vortex.

In the simulations, a reduced form of the residual-based stabilization^31^ is used,

17 $$\begin{aligned} SD_\delta (u,p;v,q)=(\delta _1(\bar{u}\cdot \nabla )u,(\bar{u}\cdot \nabla )v) + (\delta _1 \nabla p,\nabla q) + (\delta _2\nabla \cdot u, \nabla \cdot v), \end{aligned}$$

where *v* and *q* are the velocity and pressure test functions, respectively, $(\cdot , \cdot )$ are bilinear forms, $\bar{u}$ is a linearized velocity, and the stabilizations parameters $\delta _1$ and $\delta _2$ are given by

18 $$\begin{aligned} \delta _1 = \frac{\kappa _1 \sqrt{k^{-2} + |u|^2/h^2 )}}{2}, \quad \delta _2 = \kappa _2h, \end{aligned}$$

where *k* is the time step size, *h* is the local mesh size, and $\kappa _1=4.0$ and $\kappa _2=2.0$ are constants chosen to achieve a satisfactory stabilization.

The numerical method was implemented using the library Dolfin-HPC for finite element computation, based on the FEniCS project^35,36^. The simulations were run on the supercomputer Dardel at the PDC Center for High Performance Computing at KTH Royal Institute of Technology^37^. Four nodes were used, each with two AMD EPYC^TM^ Zen2 2.25 GHz 64-core processors. The flow was simulated to a final time $T=100$ with a timestep $k=5.2\times 10^{-3}$, which took in total just over 80 hours to run.

### Data analysis

Velocity and pressure fields were computed directly in the Dolfin-HPC simulation, as was the triple decomposition, using our established algorithm^20^. Visualization and computation of the energy spectrum was done in Paraview, using Python with the package NumPy for the Fourier transform.

## Data Availability

Dolfin-HPC is available as an open source C++ library. The source code for the simulations, along with the mesh file, as well as all results files, and Paraview state files used for post-processing, are available upon request from the second author.
